# Danthron Attenuates Intestinal Inflammation by Modulating Oxidative Stress via the EGFR-PI3K-AKT and Nrf2-HO-1 Pathways

**DOI:** 10.3390/antiox15020157

**Published:** 2026-01-23

**Authors:** Chujun Ni, Haiqing Liu, Haiyang Jiang, Zexing Lin, Kangjian Wu, Runnan Wang, Huan Yang, Weijie Li, Chaogang Fan, Yun Zhao

**Affiliations:** 1 Comprehensive Laboratory, Nanjing BenQ Medical Center, The Affiliated BenQ Hospital of Nanjing Medical University, Nanjing 210019, China; 60406070e@ntnu.edu.tw (C.N.); haiqing.liu@benqmedicalcenter.com (H.L.); haiyang@stu.njmu.edu.cn (H.J.); linzexing@stu.njmu.edu.cn (Z.L.); cherryrunnan@stu.njmu.edu.cn (R.W.); njyanghuan@stu.njmu.edu.cn (H.Y.); 2 The Clinical Translational Research Center for Surgical Infection and Immunity of Nanjing Medical University, Nanjing 210019, China; 3 Department of General Surgery, Nanjing BenQ Medical Center, The Affiliated BenQ Hospital of Nanjing Medical University, Nanjing 210019, China; kangjianwu2015@njmu.edu.cn (K.W.); liweijie@stu.njmu.edu.cn (W.L.)

**Keywords:** IBD, Danthron, oxidative stress, macrophage, epithelium

## Abstract

Inflammatory bowel disease (IBD) is characterized by excessive oxidative stress, mitochondrial dysfunction, and persistent activation of pro-inflammatory signaling pathways. Danthron, a natural anthraquinone derivative from rhubarb, has been reported to possess anti-inflammatory and antioxidant properties, yet its regulatory mechanisms in intestinal inflammation remain unclear. In this study, we combined network pharmacology, transcriptomic profiling, cell-based assays, intestinal organoids, and a dextran sulfate sodium (DSS)-induced colitis model to determine the protective effects of Danthron against oxidative injury. Integrated target prediction and RNA-seq analysis identified EGFR–PI3K–AKT and Nrf2–HO-1 as key signaling axes modulated by Danthron. In macrophages and intestinal epithelial cells, Danthron markedly suppressed LPS- or H_2_O_2_-induced ROS accumulation, lipid peroxidation, and mitochondrial membrane potential collapse, while restoring superoxide dismutase activity and reducing malondialdehyde levels. Danthron also inhibited M1 macrophage polarization, preserved epithelial tight-junction proteins, and maintained transepithelial electrical resistance. CETSA, DARTS, and molecular docking confirmed direct engagement of Danthron with components of both the EGFR–PI3K–AKT and Nrf2–HO-1 pathways. In vivo, Danthron significantly ameliorated DSS-induced colitis, reducing inflammatory cytokines, epithelial apoptosis, oxidative stress, and myeloid cell infiltration while improving mucosal architecture and enhancing organoid regenerative capacity. These findings demonstrate that Danthron exerts potent antioxidant and anti-inflammatory effects through coordinated inhibition of EGFR–PI3K–AKT signaling and activation of the Nrf2–HO-1 axis, suggesting its promise as a multi-target therapeutic candidate for IBD.

## 1. Introduction

Inflammatory bowel disease (IBD) refers to a group of chronic idiopathic gastrointestinal disorders whose etiology has not been fully clarified [1]. These conditions are characterized by complex interactions among host immune responses, genetic predispositions, and environmental factors, and primarily include Crohn’s disease (CD) and ulcerative colitis (UC) [2,3]. Clinically, IBD manifests as abdominal pain, distension, diarrhea, and the passage of bloody, mucoid, or mucopurulent stools [4]. Their global prevalence and incidence have demonstrated a persistent upward trajectory, and inadequate management may precipitate severe complications including colorectal cancer, conferring substantial health burdens [5,6].

IBD pathogenesis fundamentally involves compromised intestinal barrier integrity coupled with dysregulated immune activation [7,8]. As integral elements of the innate immune system, macrophages populate diverse anatomical sites [9,10]. Their phenotypic and functional heterogeneity arises from divergent cellular ontogenies and differential responses to tissue-derived signals. This plasticity manifests as distinct polarization states, conventionally defined as classically activated (M1) or alternatively activated (M2) phenotypes [11]. Furthermore, as one of the most critical components of the intestinal barrier, impairment of the structure and function of intestinal epithelial cells can often contribute to the further progression of the disease [12]. Current studies have reported that mitochondrial dysfunction and overproduction of reactive oxygen species are recognized key pathogenic mechanisms and potential therapeutic targets in IBD [13]. Our previous results also indicate that targeting mitochondrial damage and the generation of reactive oxygen species may serve as an effective strategy to control the progression of inflammatory responses and mitigate intestinal tissue damage [14,15,16].

The PI3K/AKT signaling pathway plays a crucial role in the regulation of key biological processes, including cell cycle progression, inflammatory responses, and oxidative stress [17,18]. Previous studies have demonstrated that the PI3K/AKT signaling pathway exhibits marked activation in both patients with IBD and in animal models of enteritis [19,20]. Inhibiting the activation of PI3K/AKT has emerged as a critical therapeutic target and strategy for managing ROS accumulation, modulating macrophage polarization toward the M1 phenotype, and mitigating intestinal epithelial cell injury. Meanwhile, regulating intestinal oxidative stress constitutes a key strategy in both preventing and managing IBD.

Nuclear factor erythroid 2-related factor 2 (Nrf2), a basic leucine zipper (bZIP) transcription factor, is fundamental to cellular defense against oxidative damage. It functions by controlling the expression of various antioxidant proteins, thereby ensuring redox equilibrium within the cell [21]. When oxidative stress occurs, Nrf2 separates from its inhibitor Keap1, escapes degradation, and migrates into the nucleus. Inside the nucleus, it attaches to specific DNA sequences known as antioxidant response elements (AREs), triggering the expression of protective genes like heme oxygenase-1 (HO-1). In the context of IBD, research demonstrates that higher concentrations of Nrf2 and HO-1 proteins can counteract oxidative stress and diminish the production of pro-inflammatory signaling molecules [22,23]. Through these actions, Nrf2 contributes to intestinal health by curbing inflammatory pathways and preventing damage to the mucosal lining, thereby promoting homeostasis.

Danthron, an anthraquinone compound derived from the traditional Chinese medicinal herb rhubarb, possesses diverse biological activities such as anti-inflammatory, antibacterial, and detoxifying effects [24]. Notably, it has been shown to attenuate lipid deposition and promote glucose utilization in vitro through activation of the AMPKα pathway [25,26]. In addition, emerging evidence indicates that danthron improves obesity and MAFLD by engaging the PPARα/RXRα heterodimer–adiponectin receptor 2 axis [24]. Together, these findings underscore the considerable potential of danthron in modulating inflammatory responses. However, the role of Danthron in regulating macrophage activation and the PI3K/AKT signaling pathway during IBD remains unexplored, thus highlighting a potential direction for future research.

This study used the DSS-induced colitis mouse model and LPS-stimulated macrophages to investigate the effects of Danthron on sepsis. The results demonstrate significant activation of the PI3K/AKT pathway in the intestinal tissues of DSS-induced colitis mice. Danthron effectively inhibited PI3K/AKT signaling by protecting against mitochondrial damage, reducing the generation of ROS, and suppressing its phosphorylation, consequently attenuating M1 macrophage polarization, reducing systemic inflammation, and ameliorating intestinal injury in colitis. These findings advance the mechanistic understanding of Danthron’s actions and provide a robust scientific foundation for future clinical validation of its therapeutic potential in IBD.

## 2. Materials and Methods

### 2.1. Animal Experiments

All experiments used male C57BL/6J mice (6–8 weeks old, Nanjing Medical University) housed under a 12 h light/12 h dark cycle with controlled temperature and humidity. Mice were allowed free access to water and food. All animal procedures were approved by the Institutional Animal Care and Use Committee of Nanjing Medical University (IACUC-250816).

To inducing colitis model, following a 7-day acclimation period, 20 mice were randomly assigned to four groups (*n* = 5 per group): a healthy control group (no treatment), a Danthron-only group (10 mg/kg, administered by oral gavage), a dextran sulfate sodium (DSS)-induced colitis group, and a DSS + Danthron group (10 mg/kg, oral gavage). Danthron (10 mg/kg) was administered via intraperitoneal injection on alternating days (Days 1, 3, 5, and 7) throughout the period of DSS exposure, until the experimental endpoint. This protocol was intended to assess the prophylactic effect of Danthron on the progression of colitis. The control group received distilled drinking water for 8 consecutive days, while colitis was induced in the remaining mice by administering 3% DSS in drinking water over the same period. Body weight and fecal characteristics were recorded daily. Upon conclusion of the experiment, colonic tissues were collected, measured, and carefully cleansed to remove non-tissue debris. Sections were then prepared for hematoxylin and eosin (H&E) staining analysis. A portion of the colon samples was snap-frozen and stored at −80 °C for subsequent molecular analyses.

### 2.2. Cell Lines and Cell Culture

HT-29 cells were maintained in McCoy’s 5A medium (KGL1701-500, KeyGEN BioTECH, Nanjing, China) containing L-glutamine and sodium bicarbonate, and further supplemented with 10% fetal bovine serum (FBS) (HY-T1000, MCE, Wuhan, China) and 1% penicillin-streptomycin (P/S) (KGL2303-100, KeyGEN BioTECH, Nanjing, China). The iBMDM cell line were kindly provided by Lin Lu, grown in high-glucose DMEM (KGL1208-500, KeyGEN BioTECH, Nanjing, China), also supplemented with 10% FBS and 1% P/S. All cells were kept at 37 °C in a humidified incubator with 5% CO_2_. THP-1 cells, a human monocytic line, were acquired from the Shanghai Institute of Biochemistry and Cell Biology (Shanghai, China). The cells were cultured in RPMI-1640 medium (KGL1506-500, KeyGEN BioTECH, Nanjing, China) containing 10% FBS and 1% P/S antibiotic mixture, and maintained at 37 °C in a humidified atmosphere of 5% CO_2_.

### 2.3. Network Pharmacology Screening

#### 2.3.1. Collection of the Targets

Targets of Danthron: The Canonical SMILES of Danthron were retrieved from PubChem (https://pubchem.ncbi.nlm.nih.gov/ (accessed on 23 January 2025)). Putative protein targets were then predicted using SwissTargetPrediction (http://www.swisstargetprediction.ch/ (accessed on 23 January 2025)) with species set to Homo sapiens. Predicted targets were consolidated by removing duplicates and standardizing gene names via UniProt.

IBD-related disease targets: Inflammatory bowel disease (IBD)-associated genes/proteins were retrieved from GeneCards (https://www.genecards.org/ (accessed on 23 January 2025)), OMIM (https://www.omim.org/ (accessed on 23 January 2025)), the Therapeutic Target Database (TTD, https://db.idrblab.net/ttd/ (accessed on 23 January 2025)), and DrugBank (https://go.drugbank.com/ (accessed on 23 January 2025)). Targets were restricted to Homo sapiens, and when available, relevance/score fields were exported. Target names were standardized to UniProt gene symbols using the UniProt database (https://www.uniprot.org/ (accessed on 23 January 2025)) and duplicates removed. The intersection of these curated lists was taken to generate the final IBD-related target dataset.

Overlap of Danthron- and IBD-related targets (Venn analysis): The curated Danthron target set and the IBD target set were compared using the jVenn web tool (https://jvenn.toulouse.inrae.fr/ (accessed on 23 January 2025)).

#### 2.3.2. Protein–Protein Interaction (PPI) Network Construction

PPI network construction and analysis: The intersecting targets were entered into the STRING database (https://string-db.org/ (accessed on 23 January 2025)) with the organism set to Homo sapiens. The minimum required interaction score was set to “highest confidence” (score ≥ 0.900), while all other parameters were kept at STRING defaults. Nodes without connections were hidden to obtain a connected network, and a protein–protein interaction (PPI) map was generated.

#### 2.3.3. GO and KEGG Pathway Enrichment

The intersecting targets were submitted to Metascape (https://metascape.org/gp/index.html#/ (accessed on 13 February 2025)) for GO and KEGG pathway enrichment. The organism was set to Homo sapiens; the significance threshold was fixed at *p* < 0.01, and other parameters followed Metascape defaults. Significant terms were ranked by *p*-value to examine the main pathways and biological processes.

### 2.4. Transcriptome (RNA-seq)

iBMDM cells were allocated to four groups: NC (basal medium), Danthron (20 μM, 24 h) (HY-B0923, MCE, Wuhan, China), LPS (1 μg/mL, 24 h) (L2630, Sigma-Aldrich, St. Louis, MO, USA), and LPS + Danthron (co-treatment with LPS 1 μg/mL and Danthron 20 μM for 24 h). For RNA-seq, three biological replicates per group were collected. Total RNA was extracted, strand-specific libraries were prepared, and sequencing was performed on an Illumina platform (paired-end) (San Diego, CA, USA). The differential gene was carried out on the cloud platform of omicsmart (https://www.omicsmart.com/ (accessed on 27 February 2025)).

### 2.5. Cell Survival Rate Assay

Cell viability under Danthron and H_2_O_2_ (10011218, Sinopharm Chemical Reagent, Shanghai, China) exposure was assessed using the CCK-8 kit (C6005, NCM, Suzhou, China) following the manufacturer’s instructions. Briefly, cells were seeded into 96-well plates and treated as designed. After the indicated treatments, 10 µL CCK-8 reagent was added to each well containing 100 µL medium and incubated for 1–2 h at 37 °C protected from light. Absorbance was measured at 450 nm. Viability was calculated after blank subtraction and normalization to the untreated control using:

“Cell viability (%)” = (A_”treatment” − A_”blank”)/(A_”control” − A_”blank”) × 100%.

### 2.6. Quantitative Polymerase Chain Reaction (Q-PCR)

Total RNA was isolated with TRIZOL reagent (R401-01, Vazyme, Nanjing, China) and subsequently reverse-transcribed into cDNA using a commercial reverse transcription kit (A423-01, Vazyme, Nanjing, China). Quantitative PCR was performed on a Rotor-Gene Q real-time PCR cycler (Qiagen, Venlo, The Netherlands) with SYBR Green master mix (Q711-02, Vazyme, Nanjing, China), in accordance with the manufacturer’s instructions. All primers were designed and synthesized by GenScript (Nanjing, China), with GAPDH used as an endogenous reference gene for data normalization. Relative gene expression was determined using the 2^−ΔΔCT^ method. The sequences of all primers are provided in Table 1.

### 2.7. Calcein-AM/Propidium Iodide (PI) Staining

The cell viability and cytotoxicity were conducted as previously described. Briefly, the treated cell samples were collected to be incubated with Calcein-AM and -PI (C2015M, Beyotime, Beijing, China) at 37 °C for 30 min and photographed using an inverted fluorescence microscope (Nikon Ts2R, Tokyo, Japan).

### 2.8. Flow Cytometry (M1/M2 Phenotyping)

As previously described, cells were detached with Accutase, washed with DPBS, and fixed/permeabilized using BD Cytofix/Cytoperm solution (554714, BD Biosciences, Franklin Lakes, NJ, USA, ) for 15 min at room temperature in the dark. After washing with Perm/Wash buffer, cells were stained with APC-conjugated anti-CD86 (17-0862-82, Invitrogen, Waltham, MA, USA) and PE-conjugated anti-CD206 (12-2061-82, Invitrogen, Waltham, MA, USA) for 20–30 min at 4 °C (protected from light). Samples were washed, resuspended in FACS buffer, and acquired on a flow cytometer; data were analyzed in FlowJo. For bone-marrow-derived macrophages, FITC-conjugated anti-F4/80 (11-4801-82, Invitrogen, Waltham, MA, USA) and PE-conjugated anti-CD11b (12-0112-82, Invitrogen, Waltham, MA, USA) were included to gate F4/80^+^CD11b^+^ macrophages prior to assessing CD86/CD206 expression.

### 2.9. Western Blot

The tissue and cellular samples were homogenized in RIPA lysis buffer (P0013, Beyotime, Shanghai, China) containing a protease (HY-K0010, MCE, Wuhan, China) and phosphatase (HY-K0021, MCE, Wuhan, China) inhibitor cocktail. The protein concentration of the resulting lysates was adjusted to 1.5 μg/μL. For immunoblotting, 10 μL of each sample (equivalent to 15 μg of total protein) was loaded per lane using a 15-well comb (1.5 mm thickness). Protein bands were detected using a ChemiDoc Touch Imaging System (Bio-Rad, Hercules, CA, USA) and quantitatively analyzed with Image Lab (version 6.1) software. Band intensities were normalized to GAPDH (GB11002, Servicebio, Wuhan, China) or β-tubulin (10094-1-AP, Proteintech, Rosemont, IL, USA) as the loading control. Antibody information: EGFR (66455-1-lg, Proteintech), p-EGFR (30277-1-AP, Proteintech), AKT (10176-2-AP, Proteintech), p-AKT (4060, CST, Danvers, MA, USA), PI3K (4292, CST, Danvers, MA, USA), p-PI3K (4228, CST, Danvers, MA, USA), Nrf2 (12721, CST, Danvers, MA, USA), Keap1 (8074, CST, Danvers, MA, USA), HO-1 (43966, CST, Danvers, MA, USA), Occludin (91131, CST, Danvers, MA, USA), ZO-1 (21773-1-AP, Proteintech, Wuhan, China). Signals were detected using HRP-conjugated secondary antibodies and ECL reagents. HRP-conjugated secondary antibodies information: HRP-conjugated Goat anti-Rabbit IgG (H + L) (AS014, Abclonal, Wuhan, China ), HRP-conjugated Goat anti-Mouse IgG (H + L) (AS003, Abclonal, Wuhan, China), ColorMixed Protein Marker 180 (10–180 kDa) (AM19001p, Abclonal, Wuhan, China).

### 2.10. Transepithelial Electrical Resistance (TEER)

HT-29 Cells were seeded onto permeable inserts (24-well; 5 × 10^4^ cells/insert in 400 μL apical medium; 600 μL basolateral). Treatments were applied to the apical and/or basolateral compartments as designed. After treatment, medium was refreshed every other day until a stable monolayer formed. TEER was measured with a volt–ohm meter using chopstick electrodes (Millicell ERS-2, Millipore, Burlington, MA, USA); electrodes were disinfected with 70% ethanol and rinsed with sterile PBS before each reading. TEER (Ω·cm^2^) was calculated by subtracting the blank insert resistance and multiplying by the effective membrane area. Measurements were taken at predefined time points using the same electrode, and monolayer integrity was considered adequate when TEER ≥ 400 Ω·cm^2^.

### 2.11. Immunofluorescence Staining (IF)

Following treatment, cells underwent three PBS (G4202, Servicebio, Wuhan, China) washes, fixation with 4% paraformaldehyde (G1101, Servicebio, Wuhan, China) for 15 min, and permeabilization in 0.1% Triton X-100 (P0096, Beyotime, Shanghai, China) for 20 min. After an additional three PBS rinses, samples were blocked for 1 h using 10% goat serum. Cells were then incubated overnight at 4 °C with Nrf2 primary antibody (12721, CST, Danvers, MA, USA) (5 μg·mL^−1^), ZO-1 primary antibody (21773-1-AP, Proteintech, Wuhan, China). The following day, after further PBS washing, samples were exposed to an Alexa Fluor^®^ 594-conjugated goat anti-rabbit IgG secondary antibody (1:600) (8889, CST, Danvers, MA, USA) for 1 h at room temperature, protected from light. Finally, after a final series of PBS rinses, coverslips were mounted using DAPI-containing medium (G1012, Servicebio, Wuhan, China) and visualized by fluorescence microscopy using 340 nm (DAPI, San Francisco, CA, USA) and 590 nm (Alexa Fluor^®^ 594) excitation wavelengths.

### 2.12. Measurement of MDA Production and SOD Activity

Cells were lysed using the protocol provided by the manufacturer. The lysates were centrifuged and the resulting supernatant was collected for analysis of superoxide dismutase (SOD) (S0103, Beyotime, Shanghai, China) activity and malondialdehyde (MDA) (S0131S, Beyotime, Shanghai, China) content, as indicators of antioxidant capacity and lipid peroxidation, respectively. All measurements were conducted in accordance with the manufacturer’s protocols.

### 2.13. Mitochondrial Superoxide Detection

Mitochondrial superoxide production was assessed with the MitoSOX™ Red fluorescent probe (S0061, Beyotime, Shanghai, China), which selectively localizes to mitochondria. Live cells were loaded with 5 μM MitoSOX™ Red and incubated for 15 min at 37 °C under light-protected conditions. Following a wash with pre-warmed PBS, the cells were visualized using a Carl Zeiss LSM880 confocal microscope (Oberkochen, Germany).

Following the treatment, cells in the 6-well plates were rinsed twice with PBS. Then, 2 mL of 10 μM DCFH-DA solution (S0033, Beyotime, Shanghai, China) was applied to each well, and the cells were incubated at 37 °C under 5% CO_2_ for 30 min. After two additional washes with PBS, intracellular ROS levels were measured using flow cytometry.

### 2.14. Detection of MMP by JC-10 Staining

Following the treatment period, cells in the 6-well plates were rinsed twice with PBS. Subsequently, 1 mL of JC-10 staining solution (10 μg/mL, diluted in JC-10 assay buffer) (BB41052, Bestbio, Beijing, China) was applied to each well, and the plates were incubated for 30 min at 37 °C under 5% CO_2_ in the dark. Cells were then harvested and subjected to flow cytometric analysis using a FACS Calibur system (BD, Franklin Lakes, NJ, USA).

### 2.15. Molecular Simulation

Docking was performed in AutoDock 4.2. Protein structures (RCSB PDB) were prepared by removing waters/ligands, adding polar hydrogens, and assigning Kollman charges. Danthron (PubChem) was protonated at physiological pH, energy-minimized, and assigned Gasteiger charges. AutoGrid (AutoDock 4.2, The Scripps Research Institute, La Jolla, CA, USA) was used to generate grids centered on the canonical pockets (EGFR/PI3K/AKT/Nrf2/HO-1). Docking employed the Lamarckian Genetic Algorithm with 50 independent runs per target; other parameters were default.

### 2.16. Cellular Thermal Shift Assay (CETSA)

Cells were washed with cold PBS, and lysed in ice-cold lysis buffer. After clarification (15,000× *g*, 10 min, 4 °C), equal protein aliquots of the supernatant were incubated with Danthron 20μM or DMSO (HY-Y0320, MCE, Wuhan, China) for 30 min on ice, then distributed into PCR tubes and heated for 3 min at each temperature: 37, 41, 44, 47, 50, 53, 56, 59, 63 and 67 °C. Samples were cooled on ice, centrifuged (15,000× *g*, 20 min, 4 °C) to pellet aggregated proteins, and the soluble fractions were collected for Western blot against EGFR, PI3K, AKT, Nrf2, and HO-1.

### 2.17. Drug Affinity Responsive Target Stability (DARTS)

Clarified lysates were incubated with Danthron (20 μM) for 30 min at room temperature, followed by limited proteolysis with Pronase E (HY-114158, MCE, Wuhan, China) for 15–30 min at room temperature. Reactions were terminated by adding 4× SDS-PAGE loading buffer and heating (95 °C, 5 min). Protease resistance (stabilization) of EGFR, PI3K, AKT, Nrf2, and HO-1 was assessed by Western blot.

### 2.18. Bone Marrow-Derived Macrophage (BMDM) Isolation

Femurs and tibias were aseptically harvested from mice after euthanasia. Bone ends were cut and the marrow was flushed with cold PBS using a 25-gauge needle. Cell suspensions were gently triturated, passed through a 70 μm strainer, pelleted (300× *g*, 5 min, 4 °C), treated with ACK red blood cell lysis buffer (3 min, RT) (C3702, Beyotime, Shanghai, China), and washed with PBS. Cells were resuspended in complete medium (DMEM + 10% FBS + 1% PS), counted, and seeded at 5 × 10^5^ cells/mL. For differentiation, cultures were supplemented with M-CSF (50 ng/mL) (HY-P7085, MCE, Wuhan, China) and maintained at 37 °C, 5% CO_2_; medium was refreshed on day 3. Cells reached macrophage differentiation by day 7 and were then used for downstream experiments.

### 2.19. Immunohistochemistry (IHC)

Colonic tissue sections were fixed in 4% paraformaldehyde, embedded in paraffin, and sliced into 5 μm thick sections. Antigen retrieval was performed using citrate buffer solution (pH = 6.0) and heating in a microwave oven. Non-specific binding was blocked with 5% goat serum for 30 min. Sections were incubated overnight at 4 °C with primary antibodies, followed by incubation with a secondary antibody and staining with DAB (3,3′-diamino benzidine). Hematoxylin was used for counterstaining before the sections were examined microscopically and images were captured.

### 2.20. Organoid Extraction, Culture, and Treatment

Intestinal crypts from 6–8-week C57BL/6 mice were isolated with 3 mM EDTA (4 °C, 20 min), filtered (70 µm), pelleted, and embedded in Matrigel (354234, Corning, NY, USA) domes in 24-well plates. After solidification (37 °C, 30 min), IntestCult™ OGM Mouse Basal Medium (#06005, STEMCELL, Vancouver, BC, Canada) was added and changed every other day. After 2 days, organoids were assigned to four groups: NC, DSS (10 µg/mL) (60316ES, Yeasen Biotechnology, Shanghai, China), Danthron (20 µM), and DSS + Danthron (10 µg/mL + 20 µM) for 4 days. Organoid growth was monitored by inverted microscopy (EVOS-AFM5000, Thermo, Waltham, MA, USA).

### 2.21. Statistical Analysis

All graphical presentations and statistical analyses were performed with GraphPad Prism (version 9.0). Group comparisons were conducted using an unpaired *t*-test or one-way ANOVA, as appropriate. Continuous data are expressed as mean ± standard deviation (SD). For in vitro experiments, *n* represents the number of independent biological replicates, whereas for in vivo experiments, *n* denotes the number of individual animals. Statistical significance was defined as * *p* < 0.05, ** *p* < 0.01, *** *p* < 0.001 and **** *p *< 0.0001.

## 3. Results

### 3.1. Network Pharmacology and Transcriptomic Profiling Indicate Danthron Suppresses LPS-Driven Macrophage Inflammation and Pathways Linked to Intestinal Injury

**Figure 1:**
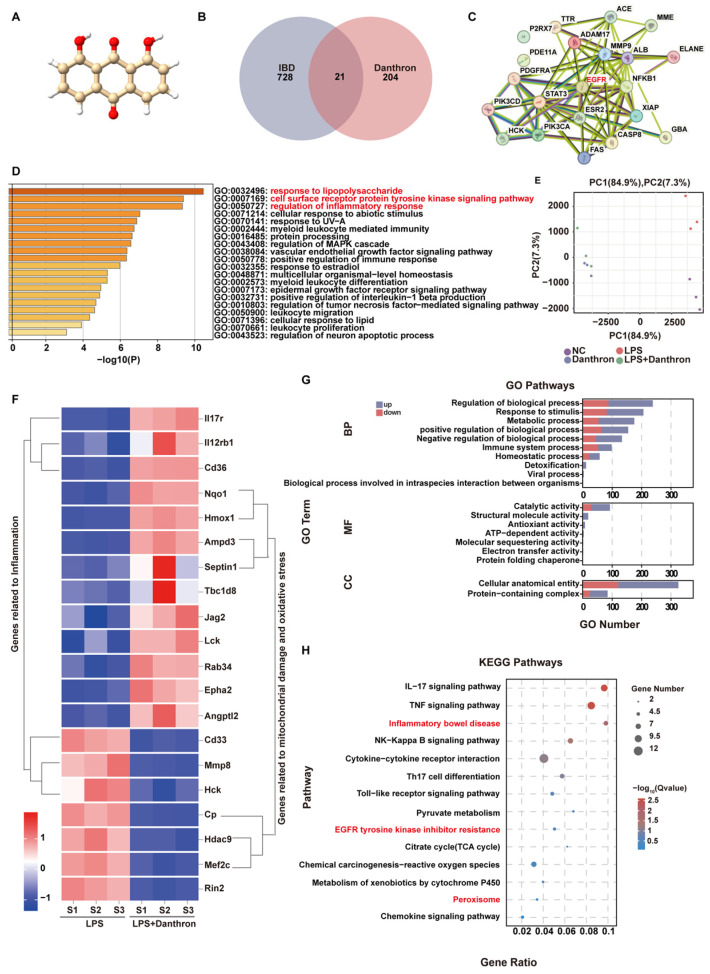
Network pharmacology and transcriptomic profiling indicate Danthron suppresses LPS-driven macrophage inflammation and pathways linked to intestinal injury. (**A**) Chemical structure of Danthron (anthraquinone derivative). (**B**) Venn diagram intersecting predicted Danthron targets with IBD-associated genes, identifying 21 shared targets. (**C**) PPI network of the 21 overlapping targets; node size reflects degree; edge thickness indicates confidence. Putative hubs (e.g., EGFR, NF-κB components, STAT3) are highlighted. (**D**) GO enrichment of the overlapping targets (Biological Process), ranked by −log10(P); selected terms include response to lipopolysaccharide, regulation of inflammatory response, and positive regulation of NF-κB transcription-factor activity. (**E**) PCA of RNA-seq profiles from macrophages after 24 h treatment: NC, LPS (100 ng/mL), and LPS (100 ng/mL) +Danthron (20μM) groups cluster separately, indicating Danthron reshapes the LPS-induced transcriptome. (**F**) Unsupervised clustering heatmap of DEGs between LPS and LPS + Danthron conditions, showing two major modules: inflammation-related genes and genes associated with mitochondrial injury/oxidative stress. Values are row-scaled (Z-score of log2 expression). (**G**) GO term enrichment of DEGs (up- vs. down-regulated sets shown separately) across BP/MF/CC categories; bars indicate gene counts. (**H**) KEGG pathway enrichment of DEGs displayed as a dot plot (gene ratio and adjusted Q value); inflammatory pathways (e.g., IL-17, TNF-α, NF-κB) and “Inflammatory bowel disease” are selectively suppressed by Danthron.).

To nominate disease-relevant targets, we first depicted the chemical structure of Danthron (Figure 1A) and intersected its predicted targets with IBD-associated genes, yielding 21 overlaps (Figure 1B). A PPI map of these overlaps revealed central hubs, including EGFR, NFKB1, and STAT3 (Figure 1C). GO enrichment of the overlapping set was dominated by terms related to innate immune activation—response to lipopolysaccharide, regulation of inflammatory response, and positive regulation of NF-κB transcription factor activity (Figure 1D).

We then profiled LPS-stimulated macrophages with or without Danthron. Principal component analysis segregated LPS from LPS + Danthron along PC1, indicating a Danthron-dependent shift in the LPS-induced transcriptome (Figure 1E). Unsupervised clustering of DEGs distinguished two major modules: one enriched for inflammation-associated genes and another for genes linked to mitochondrial damage/oxidative stress (Figure 1F). Consistently, GO analysis of DEGs highlighted processes such as inflammatory response, cytokine production, and leukocyte migration (Figure 1G). KEGG enrichment demonstrated attenuation of canonical inflammatory pathways, including IL-17, TNF-α, NF-κB, and Inflammatory bowel disease (Figure 1H).

Together, the network-pharmacology predictions and RNA-seq data indicate that Danthron reshapes LPS-driven inflammatory programs while dampening pathways associated with epithelial injury, supporting its potential utility in IBD.

### 3.2. Danthron Suppresses Macrophage Inflammatory Activation and Preserves Intestinal Epithelial Viability and Barrier Integrity

**Figure 2:**
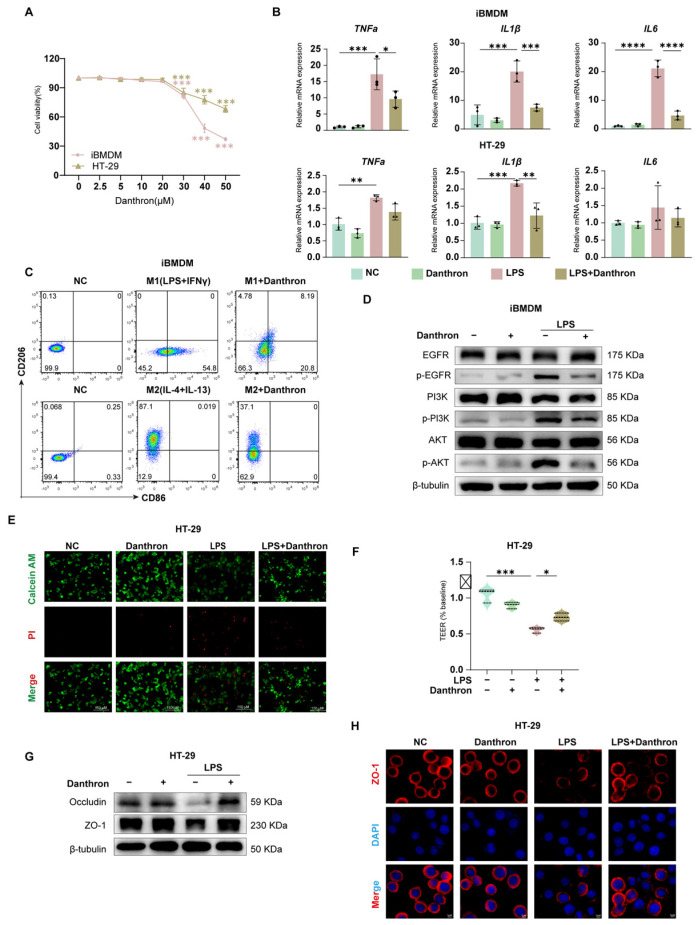
Danthron suppresses macrophage inflammatory activation and preserves intestinal epithelial viability and barrier integrity. (**A**) Cell viability of iBMDMs and HT-29 cells exposed to increasing concentrations (0, 2.5, 5, 10, 20, 30, 40, 50 μM) of Danthron for 24 h, measured by CCK-8; viability is expressed relative to vehicle. (**B**) qPCR analysis of pro-inflammatory cytokines in iBMDMs and HT-29 cells treated for 24 h under the indicated conditions: NC, Danthron alone (20 μM), LPS (100 ng/mL), and LPS (100 ng/mL) + Danthron (20 μM). Danthron markedly blunts LPS-induced cytokine transcription. (**C**) Flow-cytometric assessment of macrophage polarization. Upper row: M1 induction (LPS (100 ng/mL) +IFN-γ (10 ng/mL)) for 24 h increases CD86 and reduces CD206; Danthron attenuates the M1 phenotype. Lower row: M2 induction (IL-4 (20 ng/mL) + IL-13 (20 ng/mL)) for 24 h elevates CD206; Danthron does not impede M2 features and modestly enhances CD206. (**D**) Immunoblotting of EGFR/PI3K/AKT signaling in iBMDMs. LPS (100 ng/mL) for 24 h elevates phospho-EGFR, phospho-PI3K, and phospho-AKT; Danthron (20 μM) reduces pathway activation. β-tubulin serves as a loading control.(**E**) Live/dead staining of HT-29 monolayers using Calcein-AM (live, green) and propidium iodide (dead, red). LPS (100 ng/mL) for 24 h increases cell death, which is reduced by Danthron (20 μM). Scale bars, 150 μm. (**F**) TEER of HT-29 monolayers normalized to baseline. LPS (100 ng/mL) for 24 h disrupts barrier function, whereas Danthron (20 μM) preserves TEER. (**G**) Immunoblotting of tight-junction proteins in HT-29 cells. LPS (100 ng/mL) for 24 h diminishes Occludin and ZO-1, which are restored by Danthron (20 μM). (**H**) Immunofluorescence of ZO-1 in HT-29 monolayers (red) with nuclear counterstain (DAPI, blue). Danthron (20 μM) for 24 h maintains continuous junctional ZO-1 disrupted by LPS (100 ng/mL). Scale bars, 5 μm. Statistical significance was determined by one-way ANOVA followed by Tukey’s post hoc test. * *p* < 0.05, ** *p* < 0.01, *** *p* < 0.001, **** *p *< 0.0001.

We first established a working concentration range for Danthron. Across iBMDM, THP-1 and HT-29 cells, Danthron exhibited minimal cytotoxicity within the experimental window used for subsequent assays (Figure 2A and Figure S1A). In LPS-stimulated iBMDMs and THP-1, Danthron significantly reduced transcription of TNF-α, IL-1β, and IL-6, and a similar attenuation was observed in HT-29 cells (Figure 2B and Figure S1B). Flow cytometry showed that Danthron curtailed M1 polarization (reduced CD86 with LPS + IFN-γ) while preserving the IL-4/IL-13-driven M2 phenotype (CD206) (Figure 2C).

In epithelial monolayers, Danthron improved cell survival under LPS challenge (Calcein-AM/PI) (Figure 2D), preserved transepithelial electrical resistance (TEER) (Figure 2F), and restored tight-junction proteins. Immunoblotting demonstrated recovery of Occludin and ZO-1 abundance (Figure 2G), and immunofluorescence confirmed maintenance of continuous junctional ZO-1 that was disrupted by LPS (Figure 2H).

Mechanistically, Danthron dampened proximal inflammatory signaling in macrophages: LPS-induced phosphorylation of EGFR, PI3K, and AKT was reduced by Danthron without altering total protein levels (Figure 2E and Figure S1H). Collectively, these data show that Danthron blunts macrophage inflammatory activation and protects epithelial viability and barrier integrity during LPS exposure.

### 3.3. Danthron Mitigates Oxidative Stress and Preserves Mitochondrial Function—Partly Through Nrf2/HO-1 Activation in LPS-Challenged Macrophages and Intestinal Epithelial Cells

**Figure 3:**
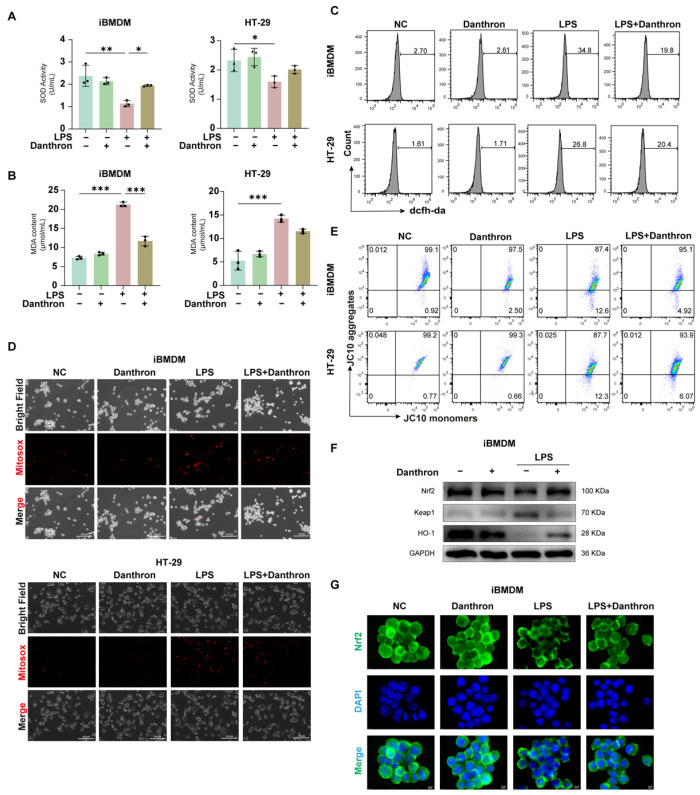
Danthron mitigates oxidative stress and preserves mitochondrial function–partly through Nrf2/HO-1 activation in LPS-challenged macrophages and intestinal epithelial cells. (**A**) SOD activity in iBMDMs and HT-29 cells treated for 24 h under the indicated treatments (NC, Danthron (20 μM), LPS (100 ng/mL), LPS (100 ng/mL) + Danthron (20 μM)). LPS reduces antioxidant capacity, which is restored by Danthron. (**B**) MDA content as an index of lipid peroxidation. Danthron (20 μM)) markedly attenuates the LPS (100 ng/mL)-induced increase in MDA in both cell types after 24 h treatment. (**C**) Flow-cytometric measurement of cellular ROS using DCFH-DA. Histograms show that after 24 h stimulation, Danthron (20 μM) lowers the LPS (100 ng/mL)-evoked ROS surge in iBMDMs and HT-29 cells. (**D**) MitoSOX fluorescence microscopy of mitochondrial superoxide. LPS (100 ng/mL) for 24 h elevates MitoSOX signals, while Danthron (20 μM) reduces mitochondrial ROS in both iBMDMs (top panel) and HT-29 cells (bottom panel). Scale bars, 300 μm. (**E**) Mitochondrial membrane potential (ΔΨm) assessed by JC-10. Quadrant plots depict aggregates (high ΔΨm) versus monomers (low ΔΨm); LPS (100 ng/mL) for 24 h lowers ΔΨm (decreased aggregates/increased monomers), which is partially rescued by Danthron (20 μM) in both cell types. (**F**) Immunoblotting of Nrf2 pathway components in iBMDMs. Danthron (20 μM) increases Nrf2 and HO-1 with a concomitant reduction in Keap1 in LPS (100 ng/mL)-treated cells after 24 h treatment. GAPDH is the loading control. (**G**) Immunofluorescence of Nrf2 (green) with nuclear counterstain (DAPI, blue) in iBMDMs. LPS (100 ng/mL) for 24 h impairs Nrf2 nuclear accumulation, whereas Danthron (20 μM) promotes Nrf2 nuclear localization under LPS challenge. Scale bars, 5 μm. Statistical significance was determined by one-way ANOVA followed by Tukey’s post hoc test. * *p* < 0.05, ** *p* < 0.01, *** *p* < 0.001.

We next examined whether Danthron restrains LPS-evoked oxidative injury. In iBMDMs, THP-1 and HT-29 cells, LPS reduced SOD activity and increased MDA, whereas Danthron significantly restored SOD and lowered MDA in both cell types (Figure 3A,B and Figure S1C,D). Consistently, total intracellular ROS measured by DCFH-DA rose sharply with LPS and was diminished by Danthron (Figure 3C and Figure S1F). Microscopy with MitoSOX further showed that Danthron reduced mitochondrial superoxide signals that were prominent after LPS (Figure 3D and Figure S1E). In parallel, JC-10 assays indicated that LPS collapsed the mitochondrial membrane potential (decrease in aggregates/increase in monomers), which was partially rescued by Danthron in macrophages and epithelial cells (Figure 3E and Figure S1G).

To probe antioxidant signaling, we profiled the Nrf2 pathway in iBMDMs. Danthron increased Nrf2 and HO-1 abundance while decreasing Keap1 under LPS challenge (Figure 3F and Figure S1I), and immunofluorescence revealed enhanced Nrf2 nuclear accumulation in Danthron-treated cells compared with LPS alone (Figure 3G and Figure S1J). Together, these data indicate that Danthron attenuates LPS-induced oxidative stress and preserves mitochondrial function, accompanied by activation of the Nrf2/HO-1 axis.

### 3.4. Danthron Binds and Stabilizes EGFR–PI3K–AKT and Nrf2–HO-1 Signaling Nodes

**Figure 4:**
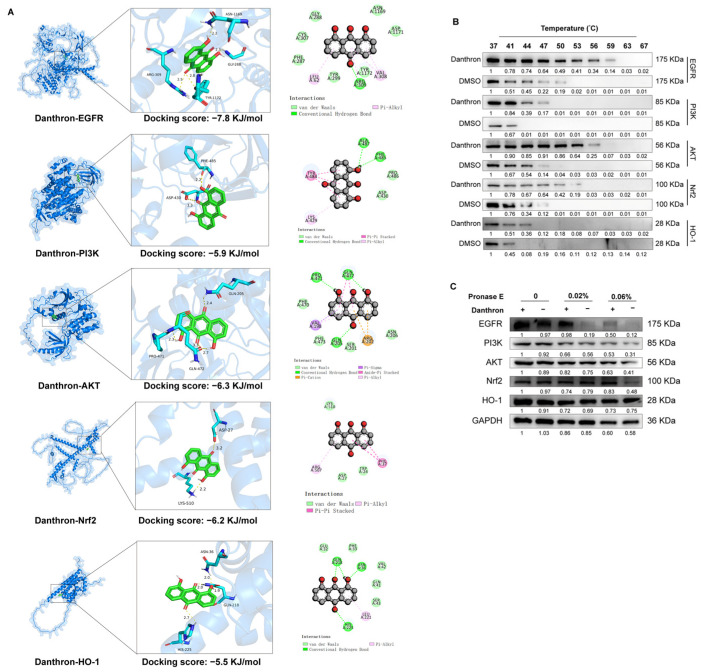
Danthron binds and stabilizes EGFR–PI3K–AKT and Nrf2–HO-1 signaling nodes. (**A**) Molecular docking models for Danthron bound to EGFR, PI3K, AKT, Nrf2, and HO-1. Insets show the ligand pose within the predicted binding pocket with representative hydrogen-bond and hydrophobic contacts. Docking scores (kJ/mol) are indicated for each target (EGFR, −7.8; PI3K, −5.9; AKT, −6.3; Nrf2, −6.2; HO-1, −5.5), suggesting favorable interactions. (**B**) CETSA. Cells (iBMDMs) were treated with Danthron (20 μM) or vehicle (DMSO) for 24 h, lysed, and aliquots were heated at the indicated temperatures (37, 41, 44, 47, 50, 53, 56, 59, 63, 67 °C) before immunoblotting. Danthron increased the thermal stability (retained signal at higher temperatures) of EGFR (175 kDa), PI3K (85 kDa), AKT (56 kDa), Nrf2 (~100 kDa), and HO-1 (28 kDa), consistent with target engagement. (**C**) DARTS. Lysates pre-incubated with Danthron (20 μM) or vehicle were digested with Pronase E (0, 0.02%, 0.06%) and probed by immunoblotting. Danthron protected EGFR, PI3K, AKT, Nrf2, and HO-1 from proteolysis in a protease-dose–dependent manner, further supporting direct interaction. GAPDH served as a loading control.

We next evaluated whether Danthron directly engages proteins that coordinate inflammatory and antioxidant programs. Molecular docking predicted favorable binding poses for Danthron within the catalytic/ligand-binding pockets of EGFR, PI3K, AKT, Nrf2, and HO-1, supported by hydrogen-bond (estimated docking energies ~−7.8 to −5.5 kJ/mol; Figure 4A). In cellular thermal shift assays (CETSA), Danthron treatment conferred the strongest thermal stabilization to EGFR and NRF2. This was demonstrated by the relative preservation of band intensity for these proteins on immunoblots at higher temperatures, indicating their increased stability in the presence of Danthron compared to the vehicle control (Figure 4B) A similar pattern was observed in drug affinity responsive target stability (DARTS) experiments, where Danthron provided the strongest protection against proteolytic digestion for EGFR and NRF2 (Figure 4C). While detectable stabilization was also noted for PI3K, AKT, and HO-1, the effects were comparatively modest. Together, these data indicate that Danthron directly engages EGFR and NRF2 within the cellular milieu, with a potentially secondary interaction profile involving other components of the EGFR–PI3K–AKT and Nrf2–HO-1 pathways.

### 3.5. Danthron Ameliorates DSS-Induced Colitis, Limits Epithelial Apoptosis, and Preserves Junctional Proteins In Vivo

**Figure 5:**
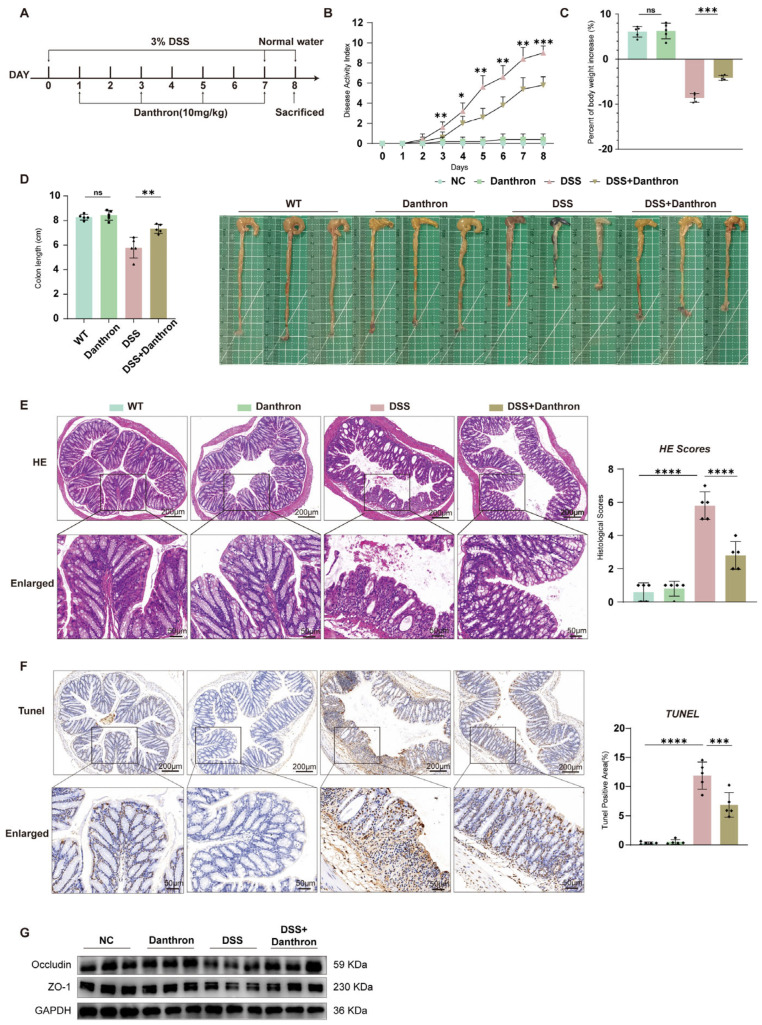
Danthron ameliorates DSS−induced colitis, limits epithelial apoptosis, and preserves junctional proteins in vivo. (**A**) Experimental scheme. Mice were administered 3% DSS in drinking water for 7 days followed by normal water; Danthron (10 mg/kg) was administered on days 1, 3, 5, and 7 during DSS exposure. Animals were sacrificed at the end of the protocol. (**B**) DAI recorded daily. 3% DSS increased DAI, whereas Danthron (10 mg/kg) significantly blunted disease progression. (**C**) Percent body−weight change at endpoint. 3% DSS caused marked weight loss that was mitigated by Danthron (10 mg/kg). (**D**) Representative colons and quantification of colon length. 3% DSS shortened the colon, which was partially restored by Danthron (10 mg/kg). (**E**) Representative H&E images of distal colon and corresponding histopathology scores. 3% DSS induced epithelial erosion, crypt loss, and inflammatory infiltrates; Danthron (10 mg/kg) reduced tissue injury. Scale bars, 200 μm (top) and 50 μm (enlarged bottom). (**F**) TUNEL staining of colon sections and quantification of TUNEL−positive cells per high−power field (HPF). 3% DSS markedly increased epithelial apoptosis; Danthron (10 mg/kg) decreased TUNEL positivity. Scale bars, 200 μm (top) and 50 μm (enlarged bottom). (**G**) Immunoblotting of tight−junction proteins from colonic tissue. 3% DSS reduced Occludin and ZO−1 abundance; Danthron (10 mg/kg) preserved their expression. GAPDH served as a loading control. Statistical significance was determined by one-way ANOVA followed by Tukey’s post hoc test. ns, not significant* *p* < 0.05, ** *p* < 0.01, *** *p* < 0.001, **** *p *< 0.0001.

Healthy C57BL/6 mice were administered 3% DSS and treated with either DMSO or danthron at a dosage of 10 mg/kg (Figure 5A). Mice treated with DSS exhibited more severe diarrhea and hematochezia compared to those receiving Danthron treatment. Daily monitoring included body weight changes, stool consistency, and the presence of hematochezia. The Disease Activity Index (DAI) score, a composite measure reflecting the severity of colitis, was significantly higher in the DSS group than in the negative control (NC) group. In comparison with the DSS group, Danthron treatment markedly reduced the DAI score (Figure 5B).

Consistent with the DAI results, Danthron treatment significantly ameliorated DSS-induced body weight loss (Figure 5C) and prevented the DSS-induced shortening of colon length (Figure 5D), further confirming the protective role of Danthron against colitis.

Histopathological analysis by H&E staining revealed that DSS challenge caused severe inflammatory cell infiltration and impaired intestinal barrier integrity. In contrast, Danthron treatment substantially reduced these pathological changes (Figure 5E). Furthermore, TUNEL staining showed a significant increase in apoptotic cells in the colonic tissues of DSS-treated mice, which was effectively suppressed by Danthron administration (Figure 5F), suggesting that Danthron inhibits DSS-induced epithelial cell apoptosis.

Finally, to assess the effect of Danthron on intestinal epithelial barrier function, we examined key markers. The results demonstrated that Danthron treatment significantly mitigated the impairment of the intestinal epithelial barrier caused by DSS (Figure 5G). Collectively, these findings indicate that Danthron effectively attenuates DSS-induced colitis by reducing disease severity, inflammation, apoptosis, and preserving epithelial barrier function.

### 3.6. Danthron Reduces Colonic Inflammation and Oxidative Stress, Restrains Myeloid Activation, and Modulates EGFR–PI3K–AKT and Nrf2–HO-1 Signaling in DSS Colitis

**Figure 6:**
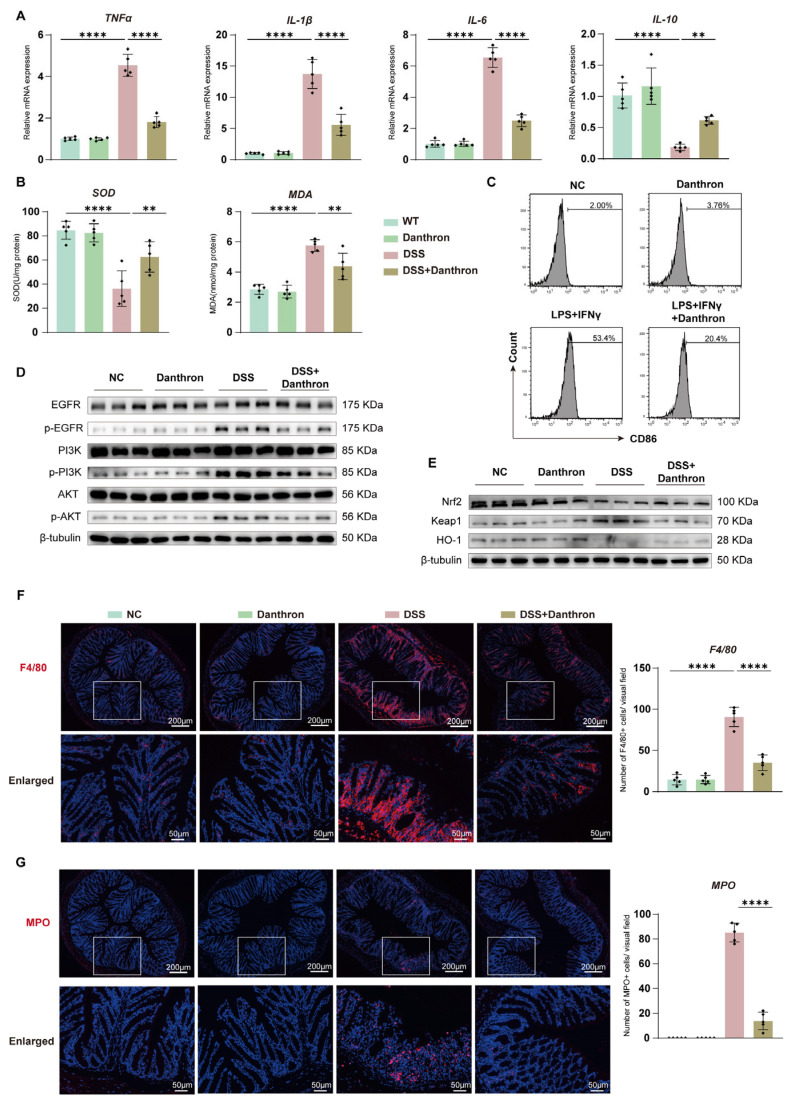
Danthron reduces colonic inflammation and oxidative stress, restrains myeloid activation, and modulates EGFR–PI3K–AKT and Nrf2–HO-1 signaling in DSS colitis. (**A**) Colonic mRNA expression of pro-inflammatory cytokines (TNF-α, IL-1β, IL-6) and the anti-inflammatory cytokine IL-10 in the indicated groups (NC, Danthron (10 mg/kg), 3% DSS, 3% DSS + Danthron (10 mg/kg)). DSS robustly elevates inflammatory transcripts, which are attenuated by Danthron; IL-10 is preserved/increased with Danthron. (**B**) Antioxidant/oxidative-damage indices in colon tissue: SOD activity and MDA content. 3% DSS lowers SOD and increases MDA; Danthron (10 mg/kg) reverses both trends. (**C**) Flow-cytometric histograms of CD86 in colon-derived macrophages following ex vivo stimulation (LPS (100 ng/mL) + IFN-γ (10 ng/mL)) with or without Danthron (20 μM). Danthron reduces CD86 upregulation. (**D**) Immunoblotting of EGFR–PI3K–AKT signaling in colonic lysates. 3% DSS increases phosphorylated EGFR, PI3K, and AKT, whereas Danthron (10 mg/kg) suppresses pathway activation. β-tubulin, loading control. (**E**) Immunoblotting of Nrf2 pathway proteins. Danthron (10 mg/kg) elevates Nrf2 and HO-1 while decreasing Keap1 in 3% DSS colons. β-tubulin, loading control. (**F**) Immunofluorescence staining of F4/80 (macrophages) in distal colon with quantification (right). DSS increases F4/80^+^ cells, which is reduced by Danthron. Scale bars, 200 μm (top) and 50 μm (enlarged). (**G**) Immunostaining of myeloperoxidase (MPO, neutrophils) with quantification (right). 3% DSS markedly raises MPO^+^ cells; Danthron (10 mg/kg) lowers neutrophil accumulation. Scale bars, 200 μm (top) and 50 μm (enlarged). Statistical significance was determined by one-way ANOVA followed by Tukey’s post hoc test. ** *p* < 0.01, **** *p* < 0.0001.

We profiled inflammatory, redox, and signaling readouts in colonic tissue. DSS robustly increased mRNA levels of TNF-α, IL-1β, and IL-6, whereas Danthron significantly attenuated these transcripts and partially preserved IL-10 (Figure 6A). In parallel, Danthron increased SOD activity and lowered MDA content relative to DSS alone, consistent with a reduced oxidative burden (Figure 6B).

Flow cytometry of BMDMs showed that CD86 up-regulation after stimulation with LPS and IFN-γ was blunted by Danthron, indicating restraint of M1 activation (Figure 6C). Immunoblotting revealed that DSS heightened phosphorylation of EGFR, PI3K, and AKT; Danthron suppressed this activation without altering total protein abundance (Figure 6D). Conversely, Danthron increased Nrf2 and HO-1 with a concomitant reduction in Keap1, indicating engagement of antioxidant defenses (Figure 6E).

Immunofluorescence corroborated decreased myeloid infiltration. DSS caused marked accumulation of F4/80-positive macrophages and MPO-positive neutrophils in the distal colon, and both populations were significantly reduced by Danthron (Figure 6F–G). Collectively, Danthron dampens colonic inflammatory signaling and oxidative stress, limits myeloid accrual, and rebalances the EGFR–PI3K–AKT and Nrf2–HO-1 pathways during DSS colitis.

### 3.7. Barrier Protection by Danthron: Reinforced Epithelium and Improved Organoid Survival After DSS

**Figure 7:**
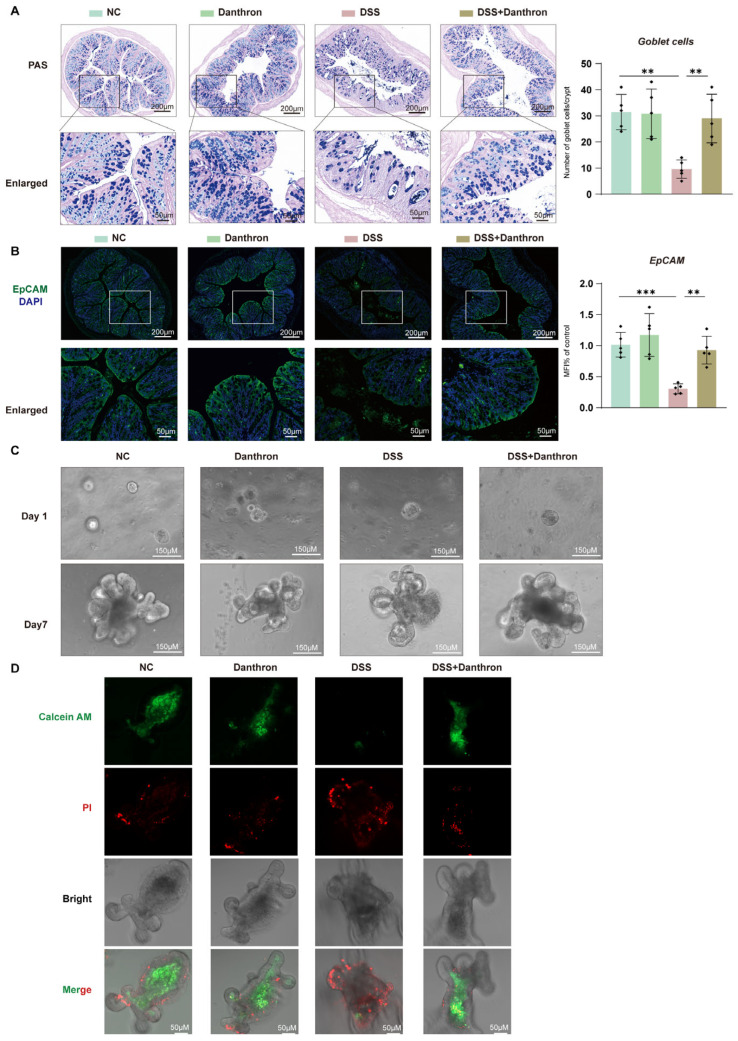
Barrier protection by Danthron: reinforced epithelium and improved organoid survival after DSS. (**A**) PAS staining of distal colon showing goblet cells (purple) and quantification per crypt. 3% DSS markedly depletes goblet cells, whereas Danthron (10 mg/kg) restores mucin-producing cells. Scale bars, 200 μm (top) and 50 μm (enlarged). (**B**) Immunofluorescence of EpCAM (green) with nuclear counterstain (DAPI, blue) and qPCR quantification of Epcam mRNA. 3% DSS reduces epithelial EpCAM signal and transcript levels; Danthron (10 mg/kg) rescues epithelial integrity. Scale bars, 200 μm (top) and 50 μm (enlarged). (**C**) Representative bright-field images of colon-derived organoids cultured ex vivo from the indicated groups on Day 1 and Day 7. Organoids were challenged with DSS (10 μg/mL) on Day 2 and continuously exposed for 4 days. DSS-derived cultures show impaired budding and growth, which are improved by Danthron (20 μM). Scale bars, 150 μm. (**D**) Live/dead staining of organoids using Calcein-AM (live, green) and propidium iodide (dead, red). DSS (10 μg/mL) increases PI-positive cells and reduces Calcein-AM signal; Danthron (20 μM) restores viability. Scale bars, 50 μm. Statistical significance was determined by one-way ANOVA followed by Tukey’s post hoc test. ** *p* < 0.01, *** *p* < 0.001.

To determine whether Danthron sustains epithelial barrier architecture during DSS injury, we first examined mucosal morphology and epithelial identity in distal colon sections. DSS exposure caused a pronounced depletion of epithelial features, with shortened/irregular crypts and attenuated epithelial signals. Danthron treatment mitigated these changes: periodic acid–Schiff (PAS) staining showed partial restoration of mucosal architecture, and EpCAM immunofluorescence revealed stronger, more continuous epithelial staining compared with DSS alone. Consistently, Epcam mRNA levels—reduced by DSS—were significantly preserved in the Danthron group (Figure 7A–B). These data indicate that Danthron maintains epithelial identity and helps stabilize the barrier compartment in vivo.

We next asked whether this protection translated into improved epithelial regenerative capacity. Colon-derived organoids established from DSS mice exhibited poor expansion and sparse budding over seven days, indicative of compromised epithelial fitness. In contrast, organoids derived from Danthron-treated mice recovered robustly, forming larger spheroids with multiple buds by Day 7 (Figure 7C). Thus, the epithelial compartment retains greater growth potential when mice receive Danthron during DSS challenge.

Finally, we assessed epithelial viability directly. Live/dead staining demonstrated that DSS organoids contained extensive propidium iodide–positive regions with minimal Calcein-AM signal, consistent with cell death. Organoids from Danthron-treated mice showed the opposite pattern—enhanced Calcein-AM fluorescence and reduced propidium iodide uptake—confirming improved epithelial survival under injurious conditions (Figure 7D). Taken together, Danthron preserves epithelial identity, sustains regenerative growth, and enhances viability following DSS injury, supporting a barrier-protective role that complements its anti-inflammatory and antioxidant effects observed in vivo.

### 3.8. Danthron Protects Macrophages and the Intestinal Epithelial Barrier from H_2_O_2_-Induced Oxidative Injury via Modulation of EGFR–PI3K–AKT Signaling

**Figure 8:**
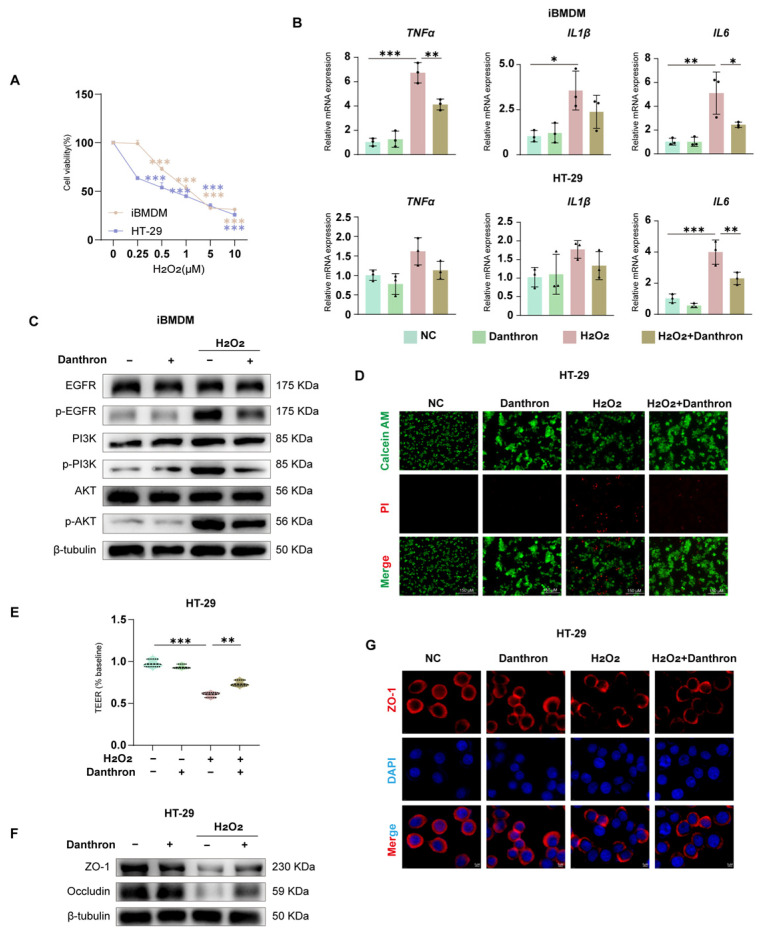
Danthron protects macrophages and the intestinal epithelial barrier from H_2_O_2_-induced oxidative injury via modulation of EGFR–PI3K–AKT signaling. (**A**) Dose–response (0, 0.25, 0.5, 1, 5, 10 μM) of H_2_O_2_ on cell viability in iBMDMs and HT-29 cells (24 h; CCK-8). (**B**) qPCR analysis of pro-inflammatory cytokines in iBMDMs (top: TNFα, IL1β, IL6) and HT-29 cells (bottom) after 24 h treatment under the indicated conditions: NC (vehicle), Danthron (20 μM), H_2_O_2_ (0.5 μM in iBMDMs and 1 μM in HT-29 cells), and H_2_O_2_ + Danthron (20 μM). Danthron blunts the H_2_O_2_-induced cytokine surge. (**C**) Immunoblotting of EGFR–PI3K–AKT signaling in iBMDMs. H_2_O_2_ (0.5 μM in iBMDMs and 1 μM in HT-29 cells) for 24 h elevates p-EGFR, p-PI3K, and p-AKT; Danthron (20 μM) suppresses pathway activation. β-tubulin, loading control. (**D**) Live/dead staining of HT-29 monolayers using Calcein-AM (live, green) and propidium iodide (dead, red). H_2_O_2_ (0.5 μM in iBMDMs and 1 μM in HT-29 cells) for 24 h increases cell death, which is reduced by Danthron (20 μM). Scale bars, 150 μm. (**E**) TEER of HT-29 monolayers normalized to baseline. H_2_O_2_ disrupts barrier function; Danthron preserves TEER. (**F**) Immunoblotting of tight-junction proteins in HT-29 cells. H_2_O_2_ (0.5 μM in iBMDMs and 1 μM in HT-29 cells) for 24 h reduces ZO-1 and Occludin abundance; Danthron (20 μM) restores their levels. β-tubulin, loading control. (**G**) Immunofluorescence of ZO-1 (red) with nuclear counterstain (DAPI, blue) in HT-29 monolayers. Danthron maintains continuous junctional ZO-1 disrupted by H_2_O_2_. Scale bars, 5 μm. Statistical significance was determined by one-way ANOVA followed by Tukey’s post hoc test. * *p* < 0.05, ** *p* < 0.01, *** *p* < 0.001.

To model oxidative injury, we first established an H_2_O_2_ dose–response, which produced a concentration-dependent loss of viability in iBMDMs and HT-29 cells over 24 h (Figure 8A and Figure S2A). Under this stress, inflammatory transcripts were strongly induced in both cell types; Danthron significantly blunted TNF-α, IL1β, and IL-6 expression in iBMDMs and HT-29 cells (Figure 8B).

Barrier readouts in epithelial monolayers showed concordant protection. Live/dead imaging revealed extensive PI uptake after H_2_O_2_ that was reduced by Danthron, with a concomitant increase in Calcein-AM fluorescence (Figure 8D). Functionally, Danthron preserved transepithelial electrical resistance (TEER) relative to H_2_O_2_ alone (Figure 8E) and restored tight-junction proteins by immunoblotting—ZO-1 and Occludin—toward control levels (Figure 8F). Immunofluorescence confirmed maintenance of continuous junctional ZO-1 that was disrupted by H_2_O_2_ (Figure 8G).

At the signaling level, H_2_O_2_ increased phosphorylation of EGFR, PI3K, and AKT in macrophages; Danthron curtailed this activation without altering total protein abundance (Figure 8C and Figure S2G). Together, these findings indicate that Danthron counteracts H_2_O_2_-evoked oxidative damage, attenuates inflammatory gene induction, and preserves epithelial barrier integrity, in part through suppression of the EGFR–PI3K–AKT pathway.

### 3.9. Danthron Mitigates H_2_O_2_-Induced Oxidative Stress and Mitochondrial Dysfunction—Partly via Nrf2/HO-1 Activation—In Macrophages and Intestinal Epithelial Cells

**Figure 9:**
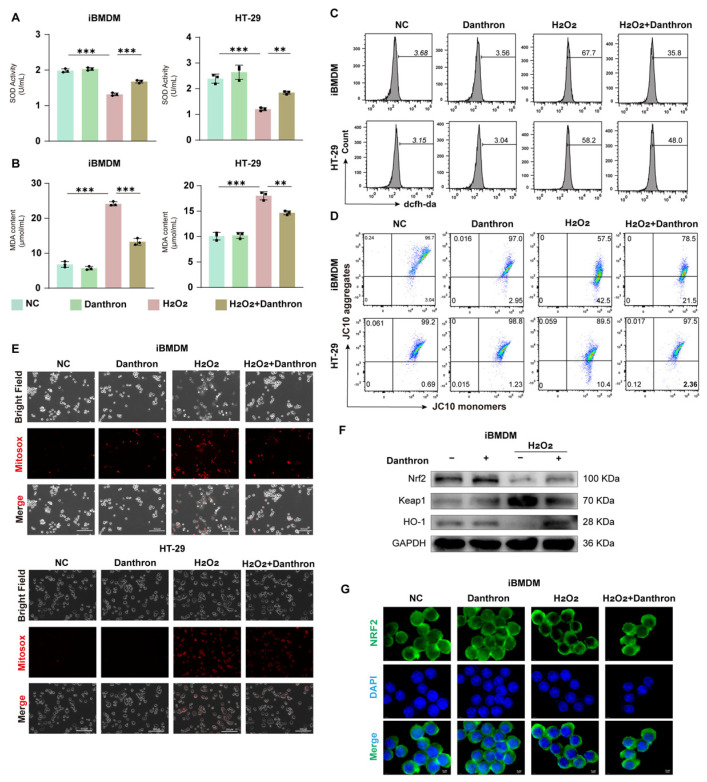
Danthron mitigates H_2_O_2_-induced oxidative stress and mitochondrial dysfunction-partly via Nrf2/HO-1 activation-in macrophages and intestinal epithelial cells. (**A**) SOD activity in iBMDMs and HT-29 cells after 24 h treatment under the indicated conditions (NC, Danthron (20 μM), H_2_O_2_ (0.5 μM in iBMDMs and 1 μM in HT-29 cells), H_2_O_2_ + Danthron (20 μM)). H_2_O_2_ reduces antioxidant capacity; Danthron restores SOD. (**B**) MDA content as an index of lipid peroxidation. Danthron (20 μM) markedly attenuates the H_2_O_2_ (0.5 μM in iBMDMs and 1 μM in HT-29 cells)-evoked increase in MDA in both cell types after 24 h treatment. (**C**) Flow-cytometric histograms of total ROS measured by DCFH-DA. Danthron (20 μM) lowers the H_2_O_2_ (0.5 μM in iBMDMs and 1 μM in HT-29 cells)-induced ROS surge in iBMDMs (**top**) and HT-29 (**bottom**) after 24 h treatment. (**D**) Mitochondrial membrane potential (ΔΨm) assessed with JC-10. Quadrant plots show aggregates (high ΔΨm) vs. monomers (low ΔΨm). H_2_O_2_ (0.5 μM in iBMDMs and 1 μM in HT-29 cells) treatment for 24 h collapses ΔΨm, which is partially rescued by Danthron (20 μM). (**E**) MitoSOX fluorescence microscopy of mitochondrial superoxide (red) with corresponding bright-field and merged images. H_2_O_2_ (0.25 μM) increases mitochondrial ROS after 24 h stimulation, whereas Danthron (20 μM) reduces the signal. Scale bars, 300 μm. (**F**) Immunoblotting of Nrf2 pathway proteins in iBMDMs. Danthron (20 μM) increases Nrf2 and HO-1 with a concomitant decrease in Keap1 under H_2_O_2_ (0.5 μM in iBMDMs and 1 μM in HT-29 cells) stress after 24 h treatment. GAPDH, loading control. (**G**) Immunofluorescence of Nrf2 (green) with nuclear counterstain (DAPI, blue) in iBMDMs. Danthron (20 μM) promotes Nrf2 nuclear accumulation during H_2_O_2_ (0.5 μM in iBMDMs and 1 μM in HT-29 cells) challenge after 24 h treatment. Scale bars, 5 μm. Statistical significance was determined by one-way ANOVA followed by Tukey’s post hoc test. ** *p* < 0.01, *** *p* < 0.001.

To test whether Danthron counteracts peroxide stress, we quantified antioxidant capacity, lipid peroxidation, ROS burden, and mitochondrial integrity in macrophages and epithelial cells. H_2_O_2_ markedly decreased SOD activity and elevated MDA in iBMDMs and HT-29 cells, whereas Danthron significantly restored SOD and reduced MDA in both models (Figure 9A,B and Figure S2B,C). Flow cytometry with DCFH-DA showed a strong ROS surge after H_2_O_2_ that was blunted by Danthron (Figure 9C and Figure S2E). Consistently, JC-10 assays indicated loss of mitochondrial membrane potential (lower aggregates, higher monomers) under H_2_O_2_, which was partially rescued by Danthron in both cell types (Figure 9D and Figure S2F). Microscopy with MitoSOX further demonstrated that Danthron reduced mitochondrial superoxide signals induced by H_2_O_2_ (Figure 9E and Figure S2D).

We next profiled the Nrf2 pathway. Immunoblotting revealed that Danthron increased Nrf2 and HO-1 with a concomitant decrease in Keap1 in H_2_O_2_-treated iBMDMs (Figure 9F and Figure S2H). Immunofluorescence confirmed enhanced Nrf2 nuclear accumulation with Danthron compared with H_2_O_2_ alone (Figure 9G and Figure S2I). Together, these findings show that Danthron mitigates H_2_O_2_-induced oxidative stress and preserves mitochondrial function in macrophages and intestinal epithelial cells, in part through activation of the Nrf2/HO-1 antioxidant program.

## 4. Discussion

In this study, we demonstrated that danthron can alleviate intestinal inflammation by inhibiting oxidative stress, through the application of network pharmacology, transcriptome sequencing, organoid culture, and related experimental approaches.

Pathological inflammation is mechanistically linked to perturbations in cellular redox dynamics, characterized by elevated free radical generation, compromised antioxidant reserves, and a consequent disruption of the oxidant-antioxidant equilibrium [27,28,29]. This oxidative milieu facilitates the accrual of ROS, inducing macromolecular damage that perpetuates a self-amplifying inflammatory cascade [30,31]. Critically, ROS function dually as both biomarkers of inflammatory activity and instigators of its escalation, underscoring the therapeutic imperative of preserving intracellular redox homeostasis [32,33]. In the pathogenesis of IBD, the complex functionality of ROS has been elucidated [34,35]. Congruent with this paradigm, our data demonstrate that aberrant intracellular ROS elevation drives concomitant M1 macrophage polarization and enterocyte apoptosis. Significantly, pharmacological intervention with Danthron attenuates both pathological processes, thereby restoring mucosal homeostasis. Fundamentally, ROS-driven M1 macrophage polarization constitutes a pervasive cellular manifestation, suggesting Danthron’s suppression of this process engages multifaceted regulatory mechanisms warranting further mechanistic elucidation. Concurrently, ROS orchestrate diverse cell death modalities and inflammatory signaling cascades, exemplified through ROS-dependent palmitoylation of gasdermin D (GSDMD) inducing pyroptosis and potentiation of ferroptosis pathways [35,36]. This paradigm positions Danthron as a pleiotropic inhibitor that coordinately disrupts inflammatory-ROS amplification circuits in intestinal inflammation. This study employed an acute H_2_O_2_ challenge as a well-defined model to investigate the direct antioxidant properties of Danthron. Although such an acute oxidative insult differs from the chronic, immune-mediated redox imbalance characteristic of inflammatory bowel disease (IBD), it serves as a valuable and widely utilized tool for elucidating fundamental cytoprotective mechanisms. Importantly, the protective effect of Danthron observed in this model was not an isolated finding but aligned coherently with its efficacy in more pathophysiologically relevant contexts. In both LPS-stimulated macrophages and the DSS-induced colitis model, Danthron similarly attenuated oxidative damage while concurrently suppressing pro-inflammatory signaling—such as via the EGFR–PI3K–AKT axis—and enhancing endogenous defense pathways, including the Nrf2–HO-1 axis. This consistent activity across experimental systems supports a multimodal mechanism in which Danthron targets both oxidative stress and its upstream inflammatory drivers, a combination highly relevant to IBD pathogenesis. Nevertheless, we acknowledge that acute H_2_O_2_ exposure does not fully replicate the persistent, low-grade oxidative stress or the complex cellular interactions present in human IBD. Future studies utilizing chronic stress models or patient-derived systems will be important to further elucidate the sustained efficacy and translational potential of Danthron.

Danthron mediates its therapeutic actions by targeting key regulatory components within the EGFR–PI3K–AKT and Nrf2–HO-1 signaling pathways. Using integrated network pharmacology and transcriptomic profiling, we identified Danthron-responsive targets that are strongly linked to IBD pathogenesis. Further analysis revealed that both the EGFR–PI3K–AKT and Nrf2–HO-1 pathways play critical roles in modulating intestinal damage and macrophage-driven inflammatory responses [19,20,22,23]. Indeed, it is well-established that under conditions of intestinal inflammatory damage and ROS accumulation, the EGFR-PI3K-AKT signaling pathway is markedly activated, thereby mediating the upregulation of downstream inflammatory factors [37,38,39]. Corroborating this mechanism, downregulation of AKT1 protein expression and inhibition of the PI3K/AKT signaling pathway effectively ameliorate symptoms of IBD in mouse models. Our findings indicate that under LPS and H_2_O_2_ stimulation, Danthron effectively suppresses the activation of the EGFR–PI3K–AKT signaling pathway and curtails the excessive production of downstream pro-inflammatory mediators, suggesting its broad anti-inflammatory potential in the context of IBD. Consistent with this, in a DSS-induced colitis model, we observed that danthron significantly ameliorated DSS-driven impairment of the intestinal epithelial barrier and reduced the release of pro-inflammatory cytokines (e.g., IL-1β, TNF-α, and IL-6), while also attenuating inflammatory cell infiltration into colonic tissues. Furthermore, the Nrf2-HO-1 axis serves as a critical defense mechanism against oxidative stress, and a body of research has established its essential role in mitigating intestinal damage in IBD. Activation of Nrf2-HO-1 signaling has been shown to alleviate ROS-induced inflammatory responses and cellular injury, underscoring its potential as a promising therapeutic target for IBD treatment [22,23]. Our results demonstrate that, in addition to inhibiting the EGFR-PI3K-AKT signaling pathway, Danthron activates the Nrf2-HO-1 axis, thereby enhancing antioxidant capacity in both cellular and murine models. This dual mechanism alleviates inflammation and reduces intestinal damage in vitro and in vivo. The safety and therapeutic potential of Danthron present a complex picture that warrants careful contextual interpretation. Early toxicological studies demonstrated that long-term co-administration of Danthron with the carcinogen 1,2-dimethylhydrazine (DMH) increased the incidence of colon and liver tumors in mice, whereas Danthron administration alone did not promote tumorigenesis, suggesting a context-dependent safety profile [40]. Conversely, recent investigations highlight its therapeutic promise; for instance, Danthron has been shown to ameliorate obesity and hepatic steatosis via AdipoR2-mediated dual activation of PPARα/AMPKα pathways [24]. This dichotomy underscores that the biological effects of Danthron are highly dependent on the pathological setting. Supporting this notion, our findings reveal that in intestinal inflammation, Danthron alleviates the inflammatory cascade by specifically inhibiting the EGFR signaling axis. This aligns with emerging evidence of its context-dependent mechanisms, as a separate recent study reported that in hepatocellular carcinoma, Danthron exerts anti-tumor effects by targeting the transcription factor RXRA [41]. Collectively, these observations reinforce that Danthron possesses multi-faceted biological properties—acting not as a uniformly toxic or beneficial agent, but as a compound whose efficacy and safety are intimately tied to the disease microenvironment. Our work contributes to this evolving understanding by delineating its protective, EGFR-targeted role in colitis, thereby offering a more balanced and nuanced perspective on its potential risks and therapeutic value in inflammatory bowel disease.

In the present studies, the selection of the 10 mg/kg dose for in vivo administration was guided by prior studies investigating Danthron in metabolic and inflammatory contexts, which established a precedent for its bioactive range [24,41]. To directly address potential safety concerns, a Danthron-only group was included in the experimental design. The absence of adverse effects in this group—as assessed by histopathology, body weight stability, and disease activity index—supports the safety of this dosage within the acute intervention setting of DSS-induced colitis. These findings reinforce the notion that the pharmacological profile of Danthron is closely tied to its dosing regimen and experimental context, underscoring the importance of rigorous, model-specific safety assessments even when employing previously reported doses. Future studies measuring pharmacokinetic parameters and systemic exposure will be essential to bridge these experimental doses to potential human therapeutic windows.

In summary, our findings indicate that Danthron alleviates intestinal inflammation, preserves epithelial barrier integrity, and mitigates inflammatory responses in murine models of IBD. As a potential therapeutic agent for IBD, Danthron exerts its effects primarily through suppressing hyperactivation of the EGFR–PI3K–AKT pathway, upregulating the Nrf2–HO-1 antioxidant axis, inhibiting pro-inflammatory cytokine expression, and reducing oxidative damage in intestinal epithelial cells. These results advance Danthron from empirical application toward a mechanism-driven therapy, highlighting its promise as a multi-target agent for inflammatory bowel disease and providing a conceptual framework for interdisciplinary research into traditional medicine-derived therapeutics.

## Figures and Tables

**Table 1:** List of Primers for qRT-PCR.

Gene	Forward Primer (5′ to 3′)	Reverse Primer (5′ to 3′)
*TNF-α-m*	GGTGCCTATGTCTCAGCCTCTT	GCCATAGAACTGATGAGAGGGAG
*TNF-α-h*	CTCTTCTGCCTGCTGCACTTTG	ATGGGCTACAGGCTTGTCACTC
*IL-1β-m*	GCAGCAGCACATCAACAAGA	GTTCATCTCGGAGCCTGTAGT
*IL-1β-h*	CCACAGACCTTCCAGGAGAATG	GTGCAGTTCAGTGATCGTACAGG
*IL-6-m*	TAGTCCTTCCTACCCCAATTTCC	GTTCATCTCGGAGCCTGTAGT
*IL-6-h*	AGACAGCCACTCACCTCTTCAG	TTCTGCCAGTGCCTCTTTGCTG
*IL-10-m*	CGGGAAGACAATAACTGCACCC	CGGTTAGCAGTATGTTGTCCAGC
*GAPDH-m*	CATCACTGCCACCCAGAGACTG	ATGCCAGTGAGCTTCCCGTTCAG
*GAPDH-h*	GTCTCCTCTGACTTCAACAGCG	ACCACCCTGTTGCTGTAGCCAA

## Data Availability

The original contributions presented in this study are included in the article/supplementary material. Further inquiries can be directed to the corresponding authors.

## Supplementary Materials

The following supporting information can be downloaded at: https://www.mdpi.com/article/10.3390/antiox15020157/s1, Figure S1: Danthron protects LPS-challenged macrophages by limiting oxidative stress, preserving mitochondrial function, and modulating EGFR–PI3K–AKT and Nrf2/HO-1 signaling; Figure S2: Danthron counters H_2_O_2_-induced oxidative injury in macrophages by limiting ROS, preserving mitochondrial potential, and modulating EGFR–PI3K–AKT and Nrf2–HO-1 signaling.
