# Supplementary material for: Danthron Attenuates Intestinal Inflammation by Modulating Oxidative Stress via the EGFR-PI3K-AKT and Nrf2-HO-1 Pathways
Source: Antioxidants (Basel). 2026 Jan 23;15(2):157. doi: 10.3390/antiox15020157 (PMC12937471; doi:10.3390/antiox15020157)
Supplement: Supplementary file 1 [file antioxidants-15-00157-s001.zip › antioxidants-4073351-supplementary.pdf]

Supplementary

Danthron Attenuates Intestinal Inflammation by Modulating Oxidative Stress via the EGFR-PI3K-AKT and Nrf2-HO-1 Pathways

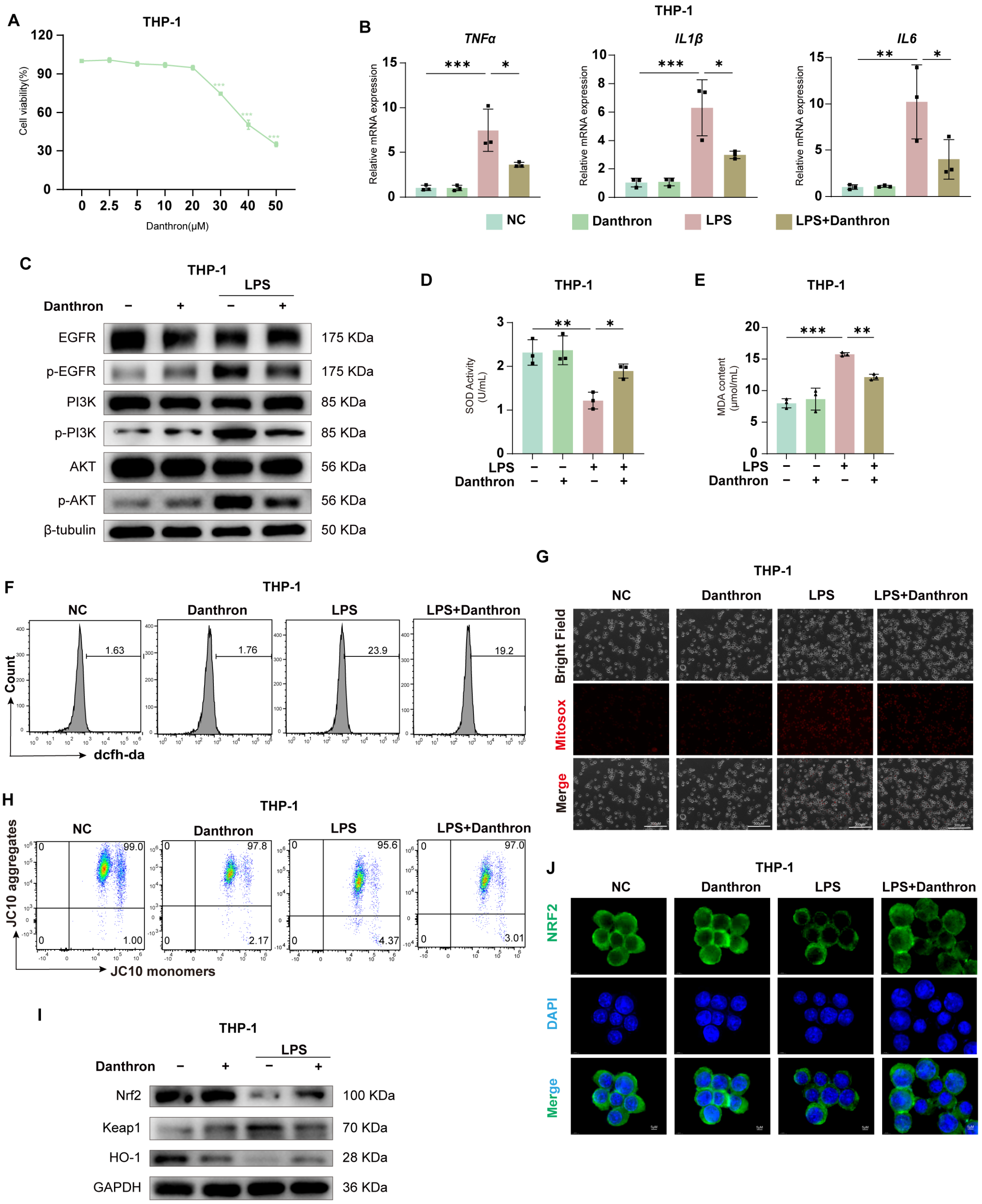
A
THP-1
Cell viability(%)
Danthron(μM)
B
THP-1
TNFα
IL1β
IL6
Relative mRNA expression
NC
Danthron
LPS
LPS+Danthron
C
THP-1
LPS
Danthron
EGFR 175 KDa
p-EGFR 175 KDa
PI3K 85 KDa
p-PI3K 85 KDa
AKT 56 KDa
p-AKT 56 KDa
β-tubulin 50 KDa
D
THP-1
SOD Activity (U/mL)
LPS
Danthron
E
THP-1
MDA content (μmol/mL)
LPS
Danthron
F
THP-1
NC
Danthron
LPS
LPS+Danthron
Count
dcfh-da
1.63
1.76
23.9
19.2
G
THP-1
NC
Danthron
LPS
LPS+Danthron
Bright Field
Mitosox
Merge
H
THP-1
NC
Danthron
LPS
LPS+Danthron
JC10 aggregates
JC10 monomers
99.0
1.00
97.8
2.17
95.6
4.37
97.0
3.01
I
THP-1
LPS
Danthron
Nrf2 100 KDa
Keap1 70 KDa
HO-1 28 KDa
GAPDH 36 KDa
J
THP-1
NC
Danthron
LPS
LPS+Danthron
NRF2
DAPI
Merge

Figure S1. Danthron protects LPS-challenged macrophages by limiting oxidative stress, preserving mitochondrial function, and modulating EGFR–PI3K–AKT and Nrf2/HO-1 signaling. (A) Cell viability of THP-1 treated with increasing concentrations(0,2.5,5,10,20,30,40,50μM) of Danthron for 24 h (CCK-8). (B) qPCR of inflammatory cytokines (TNFα, IL-1β, IL-6) in THP-1 under the indicated conditions: NC (vehicle), Danthron(20μM), LPS (100ng/mL), and LPS (100ng/mL) +Danthron(20μM). Danthron blunts the LPS-induced transcriptional surge. (C) SOD activity and (D) MDA content in THP-1. LPS (100ng/mL) for 24 h decreases SOD and elevates MDA; Danthron(20μM) reverses both trends. (E) MitoSOX fluorescence microscopy showing mitochondrial superoxide (red) with corresponding bright-field and merged images. LPS (100ng/mL) for 24 h increases mitochondrial ROS, which is reduced by Danthron(20μM). Scale bars, 300 μm. (F) Flow-cytometric histograms of total ROS (DCFH-DA). Danthron(20μM) lowers the LPS(100ng/mL)-induced ROS surge in THP-1 after 24 h treatment. (G) JC-10 assay of mitochondrial membrane potential (ΔΨm); dot plots display aggregates (high ΔΨm) versus monomers (low ΔΨm). LPS (100ng/mL) for 24 h collapses ΔΨm, partially rescued by Danthron(20μM). (H)JC-10 assay of mitochondrial membrane potential (ΔΨm). Quadrants display aggregates (high ΔΨm) vs monomers (low ΔΨm); Danthron(20μM) partially restores ΔΨm collapsed by LPS (100ng/mL) after 24 h treatment.(I) Immunoblotting of EGFR–PI3K–AKT signaling. LPS (100ng/mL) for 24 h elevates phospho-EGFR, phospho-PI3K, and phospho-AKT; Danthron(20μM) suppresses pathway activation. β-tubulin, loading control. (J) Immunoblotting of Nrf2 pathway proteins showing increased Nrf2 and HO-1 with reduced Keap1 in Danthron(20μM)-treated cells under LPS (100ng/mL) challenge after 24 h treatment. GAPDH, loading control. (K) Immunofluorescence of NRF2 (green) with nuclear counterstain (DAPI, blue) demonstrating Danthron(20μM)-enhanced nuclear accumulation of NRF2 following LPS (100ng/mL) for 24h. Scale bars, 5 μm. Statistical significance was determined by one-way ANOVA followed by Tukey's post hoc test. * $p < 0.05$, ** $p < 0.01$, *** $p < 0.001$.

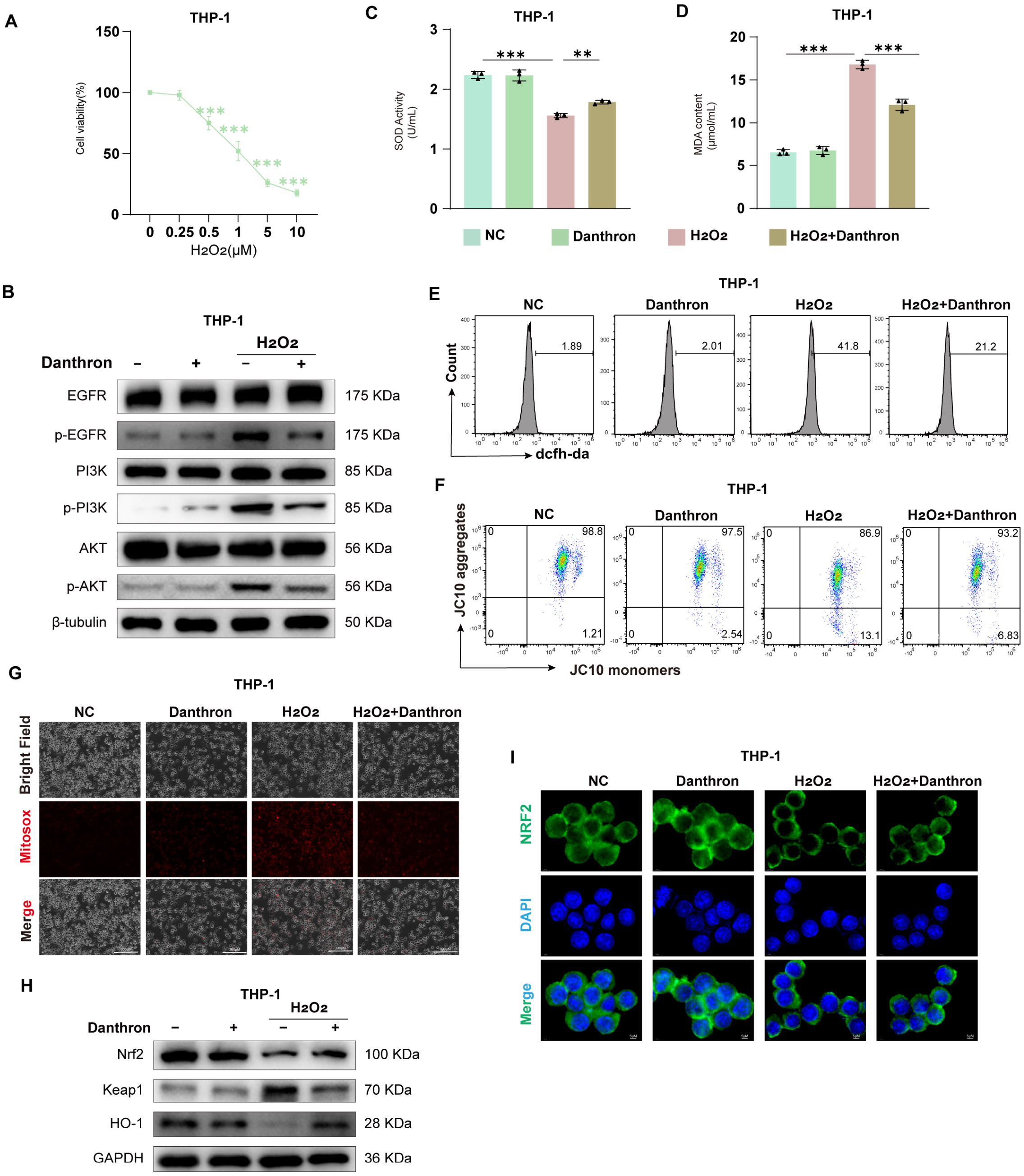
A
THP-1
Cell viability(%)
H2O2(μM)
C
THP-1
SOD Activity (U/mL)
D
THP-1
MDA content (μmol/mL)
NC
Danthron
H2O2
H2O2+Danthron
B
THP-1
H2O2
Danthron
EGFR
175 KDa
p-EGFR
175 KDa
PI3K
85 KDa
p-PI3K
85 KDa
AKT
56 KDa
p-AKT
56 KDa
β-tubulin
50 KDa
E
THP-1
Count
dcfh-da
1.89
2.01
41.8
21.2
F
THP-1
JC10 aggregates
JC10 monomers
98.8
1.21
97.5
2.54
86.9
13.1
93.2
6.83
G
THP-1
Bright Field
Mitosox
Merge
H
THP-1
H2O2
Danthron
Nrf2
100 KDa
Keap1
70 KDa
HO-1
28 KDa
GAPDH
36 KDa
I
THP-1
NRF2
DAPI
Merge

Figure S2. Danthron counters H_2O_2-induced oxidative injury in macrophages by limiting ROS, preserving mitochondrial potential, and modulating EGFR–PI3K–AKT and Nrf2–HO-1 signaling. (A) Dose–response(0,0.25,0.5,1,5,10 μ M) of H_2O_2 on THP-1 viability (24 h; CCK-8). (B) SOD) activity and (C) MDA content in THP-1 cells after 24 h treatment under the indicated conditions (NC, Danthron(20μM), H_2O_2(1μM), H_2O_2(1μM)+Danthron(20μM)). H_2O_2 decreases SOD and elevates MDA; Danthron reverses both. (D) MitoSOX fluorescence (red) showing mitochondrial superoxide with corresponding bright-field and merged images. Danthron(20μM) reduces H_2O_2(1μM)-evoked mitochondrial ROS after 24 h treatment. Scale bars, 300 μm. (E) Flow-cytometric histograms of total ROS (DCFH-DA). Danthron(20 μ M) lowers the H_2O_2(1μM)-induced ROS surge in THP-1 after 24 h treatment. (F) JC-10 assay of mitochondrial membrane potential ($\Delta\Psi$m). Quadrants display aggregates (high $\Delta\Psi$m) vs monomers (low $\Delta\Psi$m); Danthron(20 μ M) partially restores $\Delta\Psi$m collapsed by H_2O_2(1μM) after 24 h treatment. (G) Immunoblotting of EGFR–PI3K–AKT signaling. H_2O_2(1μM) for 24h increases p-EGFR, p-PI3K, and p-AKT, which are suppressed by Danthron(20 μ M). β-tubulin, loading control. (H) Immunoblotting of the Nrf2 pathway. Danthron(20 μ M) for 24h increases Nrf2 and HO-1 while decreasing Keap1 under H_2O_2(1μM) stress. GAPDH, loading control. (I) Immunofluorescence of NRF2 (green) with nuclear counterstain (DAPI, blue) in THP-1 showing Danthron(20 μ M)-enhanced NRF2 nuclear localization during H_2O_2(1μM) challenge after 24 h treatment. Scale bars, 5 μm. Statistical significance was determined by one-way ANOVA followed by Tukey's post hoc test. ** $p < 0.01$, *** $p < 0.001$.
